# Endocannabinoid system modulation for visceral abdominal pain in inflammatory bowel disease and irritable bowel syndrome: A protocol for systematic review and meta-analysis

**DOI:** 10.12688/hrbopenres.14082.2

**Published:** 2025-07-23

**Authors:** Rebecca M. Lane, Laurence J. Egan, Brian E. McGuire, Declan P. McKernan, Siobhain M. O'Mahony, David P. Finn

**Affiliations:** 1 Pharmacology and Therapeutics, School of Medicine, University of Galway, Galway, Ireland; 2 Centre for Pain Research, University of Galway, Galway, Ireland; 3 Galway Neuroscience Centre, University of Galway, Galway, Ireland; 4 Gastroenterology, Galway University Hospital, Galway, Ireland; 5 School of Psychology, University of Galway, Galway, Ireland; 6 Anatomy and Neuroscience, University College Cork and APC Microbiome Ireland, Cork, Ireland

**Keywords:** Visceral Pain, Abdominal Pain, Endocannabinoid System, IBD, IBS, Cannabis, Cannabinoid, Cannabis-Based Medicine

## Abstract

Visceral Pain is a common debilitating symptom of inflammatory bowel disease (IBD) and irritable bowel syndrome (IBS). The endocannabinoid system (ECS) is a prime target for alleviation of visceral pain, given its important role in both gastrointestinal physiology and pain. We will conduct a systematic review of randomised controlled trials (RCTs) of cannabis, cannabinoids, cannabis-based medicines (CBMs), and other ECS modulators for patients with IBD and IBS, comparing any preparation of cannabis, any cannabinoid, CBM, or other pharmacological modulator of the ECS (any dose, by any route of administration), with any control (placebo, or pharmacological / psychological / dietary intervention). We will search CENTRAL, MEDLINE, PubMed, EMBASE, and Web of Science databases, as well as the WHO International Clinical Trials Registry Platform and ClinicalTrials.gov Trials Registries, together with reference checking and citation searching, following PRISMA guidelines. Our objectives are to evaluate the benefits and harms of pharmacological modulation of the ECS for visceral abdominal pain in patients with IBD or IBS, compared to placebo or other interventions. The primary outcomes will be the proportion of people with (a) at least a 30% reduction and (b) at least a 50% reduction in pain. Secondary outcomes will include any change in pain intensity, physical and emotional functioning, fatigue and sleep measures, quality of life, gastrointestinal disease or symptom severity, and adverse effects. We will assess risk of bias in the RCTs using the Cochrane Risk of Bias 2 tool. Where there are sufficient data that are directly comparable, we will conduct meta-analyses of the results for each outcome. We will use the GRADEpro GDT tool to assess certainty of evidence for each outcome. This review will synthesise the available evidence regarding all types of ECS modulation for the treatment of visceral abdominal pain and its related comorbidities in IBS and IBD patients.

## Background

### Visceral abdominal pain in IBS and IBD

Chronic visceral abdominal pain is a cardinal diagnostic criterion of irritable bowel syndrome (IBS) ^1^, and affects up to 60% of patients with inflammatory bowel disease (IBD) irrespective of their disease activity ^2, 3^. In addition to the negative impact of chronic pain itself ^4^, the health-related quality of life of these patients is further impacted by highly prevalent comorbidities of chronic pain, including anxiety and depression ^5– 7^, and fatigue ^8– 10^. There is a sex dimorphism in visceral pain in IBS and IBD, with IBS more prevalent in females ^11^, and female sex an independent risk factor for pain in IBD ^12^. Adequate treatment of abdominal pain remains challenging ^13^. Adverse effects of commonly used analgesics (e.g., aspirin and other non-steroidal anti-inflammatory drugs, opioids) on the gastrointestinal tract are of particular concern in these patient populations ^14, 15^. Furthermore, chronic use of some analgesics, such as opioids, could lead to dependence and opioid-induced hyperalgesia ^16^. The overlap between IBS and IBD with regards visceral pain and its treatment is apparent, as is the utility of taking the lead from the IBS research, which has investigated promising avenues for pain management like anti-spasmodic (e.g. hyoscyamine), anti-convulsant (e.g. gabapentin, pregabalin) and psychopharmacological agents (e.g. tricyclic-antidepressants, selective serotonin reuptake inhibitors, serotonin-noradrenaline reuptake inhibitors) ^17, 18^. For both IBS and IBD, a holistic approach to pain management is ultimately required, one that incorporates new pharmacological therapies that target the underlying pain pathophysiology, alongside a tailored selection of dietary, psychological, and lifestyle interventions ^19, 20^.

### The Endocannabinoid System (ECS)

Modulation of the sexually dimorphic ECS ^21^, an endogenous pain control system, presents a novel avenue for management of pain and negative affective comorbidities in IBS and IBD ^22– 24^. The ECS consists of G _i/o_-coupled cannabinoid receptors 1 and 2 (CB _1_ and CB _2_) ^25– 27^, their endogenous lipid ligands ‘endocannabinoids’ anandamide (AEA) ^28^, and 2-arachydonyl glycerol (2-AG) ^29, 30^, related-lipids *N-*oleoylethanolamide (OEA), and *N-*palmitoylethanolamide (PEA) ^28– 30^ and a plethora of catabolic and anabolic enzymes, for example, fatty acid amide hydrolase (FAAH) which degrades AEA, PEA, and OEA ^31^, and monoacylglycerol lipase (MGL) which degrades 2-AG ^32^. The ECS is situated at peripheral, spinal and supra-spinal levels of the pain pathways ^22^. It is also present in neuronal and non-neuronal tissue of the gastrointestinal tract where it functions to maintain homeostasis, regulating processes such as visceral sensitivity, inflammation, motility, and secretion ^33^. Importantly, individuals with IBS and IBD have been shown to have significant alterations in their ECS compared to healthy controls, making this system a key target for treatment of their visceral pain. These alterations range from variation in genes encoding cannabinoid receptors and endocannabinoid-metabolising enzymes ^34– 36^, to differing expression of mRNA ^24, 37– 41^ and proteins related to the ECS ^37^ and differing levels of circulating endocannabinoids ^24, 38, 40^. 

### Cannabis, cannabinoids and cannabis-based medicines

There are a variety of natural and synthetic compounds that can modulate the ECS. The natural compounds are derived from *Cannabis sativa* plant material including herbal cannabis/marijuana, pharmaceutical grade medicinal cannabis, or extracts, and include individual phytocannabinoids (most notably, delta 9-tetrahydrocannabinol [THC] ^42^ and cannabidiol [CBD] ^43^). Certain extracts with specific concentrations of THC and/or CBD have regulatory approval and are classified as ‘cannabis-based medicines’ (CBMs, e.g., nabiximols ^44^, CBD ^45^). The synthetic compounds, some of which are still under development, include a variety of ECS modulators: cannabinoid receptor agonists (e.g., nabilone ^46^, dronabinol ^47^, CT-3 ^48^), cannabinoid receptor antagonists (e.g., Rimonabant ^49^), and enzyme inhibitors (e.g., PF-00457845 ^50^).

While there are a variety of compounds or formulations, few have been thoroughly tested in randomised controlled clinical trials (RCTs), and even fewer are approved by regulatory authorities for pain management, as clinical evidence for their effectiveness is limited ^51^. Study of ECS modulators is further complicated by the large variety of formulations, doses, and routes of administration available (e.g., oral, oromucosal, inhaled), making comparisons between trials difficult. Despite this, use of ECS modulators (mostly medicinal cannabis) is common amongst patients with pain, including those with IBS and IBD ^52– 54^.

### Importance of this review

Previous reviews in this area have investigated the impact of ECS modulators for IBS or IBD generally ^55– 58^, but there has been very little recognition of pain as a key outcome measure, despite the heavy burden this symptom places on patients and its relationship with emotional and physical functioning and quality of life scores. This review will place pain at the forefront, with keen attention also paid to comorbidities of chronic pain. Furthermore, previous reviews have had quite selective search terms, thus focusing on subsets of ECS modulators ^56, 57^ rather than assessing all types of ECS modulators, as this review aims to do. Finally, this review, in compiling the literature on ECS modulators across both IBS and IBD, may reveal important findings with regards similarities and differences in the response to ECS modulators between the different, yet related, populations.

## Objectives

The primary objective of this systematic review of RCTs will be to assess the effects of cannabis, cannabinoids, CBMs, and other ECS modulators for visceral abdominal pain in patients with IBS and IBD, providing estimates of effectiveness and adverse effects. The primary outcome measures will be the proportion of people with (a) at least a 30% reduction and (b) at least a 50% reduction in pain intensity (measured using a 0–10 numerical rating scale). We have chosen these specific percentage reductions as their clinical significance is supported by the Initiative on Methods, Measurement, and Pain Assessment in Clinical Trials (IMMPACT) ^59^. Key secondary objectives will include assessing the effects of cannabis, cannabinoids, CBMs and other ECS modulators on pain intensity (measured by verbal, visual, or numerical pain rating scale), pain interference, physical functioning, emotional functioning, fatigue and sleep, overall quality of life, and gastrointestinal health.

## Methods

We will follow the Preferred Reporting Items for Systematic Review and Meta-Analysis (PRISMA) guidelines ^60^.

### Criteria for considering studies

***Studies***

We will include RCTs conducted in any care setting, in any part of the world, in this systematic review.

***Participants***

We will include participants, of any age, sex, or ethnicity, with a diagnosis of IBS as defined by ROME III/IV guidelines ^1^ and/or IBD as defined by ECCO-ESGAR guidelines ^61^.

***Interventions and comparisons***

We will include any type of cannabis, cannabinoid, CBM or ECS modulator, at any dose, delivered by any route of administration, for any duration. We will also include any trials that administer cannabis, cannabinoids, CBMs or ECS modulators as part of a combination treatment, with one another or with other drugs. We will include any control; placebo, or pharmacological/psychological/dietary interventions.

We will base comparisons on the interventions grouped as follows:

- Cannabis
- Cannabinoids
- CBMs
- Other ECS modulators (e.g., cannabinoid receptor antagonists; ECS enzyme inhibitors, modulators or substrates)
- Placebo
- Alternative intervention: pharmacological, psychological, dietary

We will differentiate between interventions that have a sole aim of reducing pain intensity, and those that aim to improve IBS/IBD health outcomes more generally.

***Outcomes***

Table 1 provides a summary of the outcome measures for each of the outcome domains listed below.

**Table 1.** Summary of outcome measures.

Broad outcome grouping	Outcome domain	Outcome measures
**Pain**	Pain Intensity	Numerical pain rating scale (primary outcomes) Visual or verbal pain rating scale (secondary outcome)
**Quality of Life**	Quality of life	Quality-of-life score (e.g., short form-36, IBDQ 32, IBS-QOL)
**Physical Functioning**	Pain Interference	Interference scales of the Multidimensional Pain Inventory or Brief Pain Inventory
	Fatigue	Fatigue scores (e.g., Fatigue Severity Scales)
	Sleep	Sleep Scores (e.g., Pittsburgh Sleep Quality Index, Bergen Insomnia Scale, Mini-Sleep Questionnaire)
**Emotional Functioning**	Depression	Depression rating scale (e.g. BDI, HAM-D, PHQ-9)
	Anxiety	Anxiety rating scale (e.g., STAI, HAM-A, GAD-7)
**Gastrointestinal** **Health**	Disease severity	Standard use scale (e.g., CDAI, HBI, Mayo score), Clinical measurement (biochemical or endoscopic measures of inflammation)
	Symptoms	Symptom scales (IBS-SSS)
**Analgesic effects**	Onset	
	Duration	
**Rescue Analgesia**	Requirement for rescue analgesia	Type of rescue analgesia used Timepoint at which rescue analgesia was required Duration of rescue analgesia
	Change to analgesia used as part of usual care	Was this type of analgesia already used as part of usual care?
**Adverse Events**	Measures of Harm	Withdrawal due to serious AEs Withdrawal due to AEs Patients reporting any AE

*BDI; Beck Depression Inventory, HAM-D; Hamilton Depression Rating Scale, PHQ-9; Patient Health Questionnaire-9, STAI; State-Trait Anxiety Inventory, HAM-A; Hamilton Anxiety Rating Scale, GAD-7; Generalised Anxiety Disorder, IBDQ32; IBD Questionnaire 32, IBS-QOL; IBS Quality of Life Questionnaire, CDAI; Crohn's Disease Activity Index, HBI; Harvey-Bradshaw Index, IBS-SSS; IBS Symptom Severity Scale.*

Primary outcomes

i. The proportion of people with at least 30% reduction in pain intensity
ii. The proportion of people with at least 50% reduction in pain intensity

Secondary outcomes

i. Changes in pain intensity
ii. Change in quality of life
iii. Change in physical functioning measures (incl. pain interference, fatigue and sleep measures)
iv. Change in emotional functioning measures (e.g. anxiety, depression, mood etc.)
v. Changes in gastrointestinal health measures (incl. disease and symptom severity)
vi. Adverse events (AEs): AEs will include measures of harm, including withdrawal due to serious AEs, withdrawal because of AEs, patients reporting any AE. Following the PRISMA Harms Checklist ^62^, we will describe how AEs were addressed, how they were reported, and over what period the harm was experienced.
vii. Requirement for rescue analgesia or change in dose of analgesia taken as part of usual care.
viii. Onset and duration of analgesic effects

***Exclusion criteria***

We will exclude: (1) studies that are not RCTs (e.g. cohort studies, case-control studies, cross-sectional studies), (2) grey literature, conference abstracts or case reports, (3) studies not published in the English language, or where the full text is not available in the English language.

## Data collection and analysis

### Search methods for identification of studies

We will search the literature using a staged approach. We will search CENTRAL, MEDLINE, PubMed, EMBASE, and Web of Science databases from inception to present for peer-reviewed publications with limit to publications available in the English language (see extended data for search strategy). We will search the World Health Organisation International Clinical Trials Registry Platform and the ClinicalTrials.gov online clinical trials registries. We will conduct reference checking and citation searching of included publications to search for further trials. If we identify systematic reviews in the field, we will search their reference lists. We will not search for non-RCT studies (e.g., cohort studies, case-control studies, cross-sectional studies), grey literature or conference abstracts. We will endeavour to match selected RCTs with their trial identifier.

### Selection of studies

Two authors will independently decide which of the identified trials will be selected for screening based on their titles and abstracts. Disagreements will be resolved by discussion between the two authors, or with a third author if necessary. We will retrieve the full text of any publications or reports identified as potentially relevant. Two authors will independently screen the full texts for inclusion against eligibility criteria. Disagreements will be resolved by discussion between the two authors, or with a third author if necessary. We will include any peer-reviewed publication or online trial registration that investigated the therapeutic effects of cannabis, cannabinoids, CBMs or other ECS modulators, given by any dose or route of administration, for any duration, compared with placebo, or active pharmacological, psychological, or dietary intervention, for improving health outcomes in IBS and/or IBD patients, irrespective of reported measures. We will manage the results of our search using the Rayyan online systematic review software package (this is a proprietary tool; open access alternatives are provided in the Software Availability section). We will document our screening and selection process in sufficient detail to complete a PRISMA flow chart and ‘Characteristics of excluded studies’ table. We will include trials that are registered but not yet published as ‘awaiting classification’ and describe them separately to the included studies.

### Data extraction and management

Two authors will independently extract the following data from the included trials using a data extraction spreadsheet on Microsoft Excel:

- General information: journal, reference, corresponding author, year, country where study was conducted.
- Study characteristics: aims, design, participants enrolled per arm, enrolment method, inclusion/exclusion criteria, length of follow up (including potential sources of bias).
- Participant characteristics: age, sex, comorbidities, current medications, absence/presence of pain.
- IBS/IBD classification: diagnostic criteria/guidelines used (IBS: ROME II, III, IV; IBD: ECCO-ESGAR, AGA etc.), subtype (IBS: constipation predominant [C], diarrhoea predominant [D] or mixed symptoms [M]; IBD: Crohn’s disease [CD], ulcerative colitis [UC], indeterminate colitis [IC]), and severity.
- Intervention and comparator characteristics: type of ECS modulator/comparator, dose, duration, route of administration.
- Outcomes: we will extract any outcomes listed in the primary and secondary outcomes of this protocol.

Disagreements will be resolved by discussion between the two authors, or with a third author if necessary. We will contact study authors of trials included in the review if required to obtain further data or to confirm aspects of the data available. One author will enter all extracted data into the Review Manager (RevMan) web platform (this is a proprietary tool; open access alternatives are provided in the Software Availability section). A second author will independently check the entered data for accuracy.

### Risk of bias (quality) assessment of included studies

Two authors will independently assess the risk of bias of included trials using the Cochrane Risk of Bias Tool (RoB2) (Creative Commons Attribution-Non Commercial-No Derivatives 4.0 International License) ^63^. Disagreements will be resolved by discussion between the two authors, or with a third author if necessary. We will report our judgements with supporting quotes and justifications on the following risk of bias domains in a risk of bias table: random sequence generation (selection bias); allocation concealment (selection bias); blinding of participants and personnel (performance bias) and blinding of outcome assessment (detection bias); completeness of outcome data (attrition bias), selective outcome reporting (reporting bias), and study size (bias of small size). Our judgements on a trial being of high, low, or some concern for risk of bias will follow criteria set out in the Cochrane Handbook for Systematic Reviews of Interventions ^64^ and in the Cochrane Pain, Palliative, and Supportive Care Review Group template for intervention review ^65^.

### Synthesis methods

We will tabulate two descriptive summaries of the extracted data from individual trials: one for IBS trials, and one for IBD trials. These tables will include participant characteristics, post-treatment N, length of follow up, and descriptions of interventions, comparators, duration, and outcome measures of interest for this review, including adverse event description and frequency. We will stratify by IBS and IBD classification (IBS-C/IBS-D/IBS-M and CD/UC/IC, respectively), and intervention type (cannabis, cannabinoid, CBM, other pharmacological ECS modulator), if possible. We will narratively compare the effects of ECS modulation for outcomes relevant to both IBS and IBD populations (e.g., pain intensity, physical and emotional functioning, quality of life, fatigue, sleep, adverse events). This will enable us to identify similarities and differences between the included trials for each condition and across both conditions.

We will combine data comparing ECS modulation versus control for IBS or IBD respectively in meta-analysis if sufficient data on similar outcomes from homogenous studies are available. Two authors will independently select studies suitable for inclusion in meta-analysis, with any disagreements resolved by discussion with a third author. We will only include studies with greater than 30 participants post-treatment per group in the meta-analysis, as small sample sizes can inflate effect estimates ^51, 66^. We will use Review Manager (RevMan) for meta-analysis. We will calculate standardised mean differences with 95% confidence interval (CI) for continuous outcomes, and risk ratio with 95% CI for dichotomous outcomes. We will also calculate risk ratios with 95% confidence intervals for adverse events. We will perform statistical analysis using a random effects model to account for any within-study and between study-variation. We will assess statistical heterogeneity using the *I* ^2^ statistic. We will conduct sensitivity analysis to examine the impact of risk of bias and certainty of evidence. If the data allows, we will perform the following subgroup analysis:

i Classification of IBS: IBS-C, IBS-D, IBS-M
ii Diagnostic criteria of IBS: ROME III, ROME IV
iii Classification of IBD: CD, UC, IC
iv Intervention type
v Comparator type
vi Length of follow up
vii Treatment duration

If meta-analysis is not possible due to the nature of the data available, we will synthesize the literature narratively and will create effect direction plots for our outcomes of interest.

### Certainty of the evidence assessment (GRADE)

Two authors will assess the certainty of the evidence using the Grading of Recommendations, Assessment, Development and Evaluations (GRADE) approach, and the GRADEpro GDT tool (Standard Licence) ^67, 68^. The GRADE approach deems the certainty of evidence for each outcome to be ‘high’, ‘moderate’, ‘low’ or ‘very low’ based on 5 considerations: study limitations, unexplained heterogeneity or inconsistency, imprecision, indirectness and publication bias. Disagreements will be resolved by discussion between the two authors, or with a third author if necessary.

## Data Availability

No data are associated with this article. Open Science Framework: Systematic review of endocannabinoid system modulation for visceral abdominal pain in inflammatory bowel disease and irritable bowel syndrome. https://doi.org/10.17605/OSF.IO/PJTVU ^69^. This project contains the following extended data: Abdominal pain_Medline search_original_10 Dec 24 (search strategy for MEDLINE) Abdominal pain_free software alternatives_10 Feb 25 Data are available under the terms of the Creative Commons Zero "No rights reserved" data waiver (CC0 1.0 Public domain dedication). Open Science Framework: PRISMA-P checklist for ‘Endocannabinoid system modulation for visceral abdominal pain in inflammatory bowel disease and irritable bowel syndrome: A protocol for systematic review and meta-analysis’. https://doi.org/10.17605/OSF.IO/PJTVU ^69^. Data are available under the terms of the Creative Commons Zero "No rights reserved" data waiver (CC0 1.0 Public domain dedication).
