# Supplementary material for: Endocannabinoid system modulation for visceral abdominal pain in inflammatory bowel disease and irritable bowel syndrome: A protocol for systematic review and meta-analysis
Source: HRB Open Res. 2025 Jul 23;8:40. Originally published 2025 Mar 13. [Version 2] doi: 10.12688/hrbopenres.14082.2 (PMC12375179; doi:10.12688/hrbopenres.14082.2)
Supplement: Peer review history

# Reviewer report: Reviewer response for version 2

**DOI:** 10.21956/hrbopenres.15616.r48535

**Published:** 2025-08-23

**Authors:** Simon Erridge

**Affiliations:** 1 Imperial College London, London, England, UK

Happy with the changes made - thank you. I approve indexing the manuscript.

Is the study design appropriate for the research question?

Yes

Is the rationale for, and objectives of, the study clearly described?

Yes

Are sufficient details of the methods provided to allow replication by others?

Partly

Are the datasets clearly presented in a useable and accessible format?

Not applicable

Reviewer Expertise:

Medical cannabis and pain

I confirm that I have read this submission and believe that I have an appropriate level of expertise to confirm that it is of an acceptable scientific standard.

# Reviewer report: Reviewer response for version 1

**DOI:** 10.21956/hrbopenres.15469.r46403

**Published:** 2025-03-26

**Authors:** Colm B Collins

**Affiliations:** 1 University College Dublin, Dublin, Leinster, Ireland

This represents a really interesting study which will have considerable implications for the field going forward. This is a well-written protocol which highlights the considerable cross-disciplinary expertise that the group can bring to bear. 

 I have only a few minor suggestions.

 The abstract is already acronym-heavy, so I would consider removing the reference to IMMPACT without defining the initiative for improved accessibility.

 For discussion of endocannabinoid tone in IBD patients, consider adding a reference to Leinwand et al, J Crohns Colitis. 2017 Oct 27;11(11):1369-1380 (for receptor expression in CD patients) and Matalon et al., 2021 Front. Endocrinol (for serum endocannabinoid expression in UC/CD). 

 Consider including a reference to the chronic nature of visceral pain experienced by individuals with these diseases along with the risk of dependence and opioid-induced hyperalgesia as additional reasons for needing an alternative approach to pain management in these conditions. 

 Will indeterminate colitis or infectious colitis/gastroenteritis be included within the scope of this study, given that the former is often included as part of a catch-all of IBD patients, and the latter is the number one cause of visceral pain?

Is the study design appropriate for the research question?

Yes

Is the rationale for, and objectives of, the study clearly described?

Yes

Are sufficient details of the methods provided to allow replication by others?

Yes

Are the datasets clearly presented in a useable and accessible format?

Yes

Reviewer Expertise:

My research lab is focused on the role of cannabinoid in the regulation intestinal inflammation in IBD.

I confirm that I have read this submission and believe that I have an appropriate level of expertise to confirm that it is of an acceptable scientific standard.

# Reviewer report: Reviewer response for version 1

**DOI:** 10.21956/hrbopenres.15469.r46401

**Published:** 2025-03-22

**Authors:** Simon Erridge

**Affiliations:** 1 Imperial College London, London, England, UK

This is a nice piece of work which is well written - well done.

 A few suggestions:

 Abstract

 1. For this sentence in the abstract ((a) at least a 30% reduction and (b) at least a 50% reduction in pain intensity as defined by IMMPACT) I would drop the part which says 'as defined by IMMPACT'. I understand what you mean in terms of their clinical relevance being defined by IMMPACT but the 30/50% reduction itself is not being defined by IMMPACT. 

 Introduction

 1. As per above in your objectives just a semantic point - I would edit your objectives. The primary outcome measures of 30% / 50% reduction are not defined by IMMPACT, rather the clinical significance of these cut offs are defined by IMMPACT, so would just tweak how you have worded this.

 Methods

 1. I am assuming you are including these, but it would be helpful to define additional study parameters by which you will consider their inclusion i.e. number of participants, length of follow up etc. 

 2. It would be good to define what you will include as an other ECS modulator. Are you limiting this to PEA and similar, or are you considering other medications that may impact the endocannabinoid system. 

 3. Will you include studies which use cannabis taken recreationally? I imagine there aren't many/any, but again good to define this before you start the review.

Is the study design appropriate for the research question?

Yes

Is the rationale for, and objectives of, the study clearly described?

Yes

Are sufficient details of the methods provided to allow replication by others?

Partly

Are the datasets clearly presented in a useable and accessible format?

Not applicable

Reviewer Expertise:

Medical cannabis and pain

I confirm that I have read this submission and believe that I have an appropriate level of expertise to confirm that it is of an acceptable scientific standard, however I have significant reservations, as outlined above.
